# A comprehensive landscape of AI applications in broad-spectrum drug interaction prediction: a systematic review

**DOI:** 10.1186/s13321-025-01093-2

**Published:** 2025-09-19

**Authors:** Nour H. Marzouk, Sahar Selim, Mustafa Elattar, Mai S. Mabrouk, Mohamed Mysara

**Affiliations:** 1 Bioinformatics Group, Centre for Informatics Science (CIS), School of Information Technology and Computer Science (ITCS), Nile University, 12677, Giza, Egypt; 2 Medical Imaging and Image Processing Group, Centre for Informatics Science (CIS), School of Information Technology and Computer Science (ITCS), Nile University, 12677, Giza, Egypt

**Keywords:** Artificial intelligence, Multiple drug interactions, Drug–drug interactions, Drug–disease interactions, Drug–nutrient interactions, Machine learning, Deep learning, Graph-based method

## Abstract

In drug development, managing interactions such as drug–drug, drug–disease, and drug–nutrient is critical for ensuring the safety and efficacy of pharmacological treatments. These interactions often overlap, forming a complex, interconnected landscape that necessitates accurate prediction to improve patient outcomes and support evidence-based care. Recent advances in artificial intelligence (AI), powered by large-scale datasets (e.g., DrugBank, TWOSIDES, SIDER), have significantly enhanced interaction prediction. Machine learning, deep learning, and graph-based models show great promise, but challenges persist, including data imbalance, noisy sources, Limited explainability, and underrepresentation of certain types of interactions. This systematic review of 147 studies (2018–2024) is the first to comprehensively map AI applications across major interaction types. We present a detailed taxonomy of models and datasets, emphasizing the growing roles of large language models and knowledge graphs in overcoming key limitations. Their integration—alongside explainable AI tools—enhances transparency, paving the way for AI-driven systems that proactively mitigate adverse interactions. By identifying the most promising approaches and critical research gaps, this review lays the groundwork for advancing more robust, interpretable, and personalized models for drug interaction prediction.

**Supplementary Information:**

The online version contains supplementary material available at 10.1186/s13321-025-01093-2.

## Introduction

Drug interactions can have a significant influence on both drug efficacy and safety, leading to adverse outcomes and unexpected side effects [1]. These interactions may involve other drugs, nutrients, or pre-existing medical conditions, commonly referred to as drug–drug interactions (DDIs), drug–nutrient interactions (DNIs), and drug–disease interactions (DDSIs), respectively [2–4]. The effects of these interactions can range from severe harm—such as the nearly 175,000 fatalities and 1.25 million serious adverse events reported in 2022—to negligible influence, and in some cases, may even be beneficial [2, 5]. Global efforts have since intensified, with the International Council for Harmonisation (ICH) introducing the M12 Guideline in 2024 to standardize the evaluation of drug interactions for human use during clinical development [6]. The Food and Drug Administration (FDA) and the European Medicines Agency’s Pharmacovigilance Risk Assessment Committee (EMA’s PRAC) are enhancing drug safety communication by issuing clearer labeling guidance and conducting regular assessments of potential adverse interaction signals [7, 8].

**Fig. 1:**
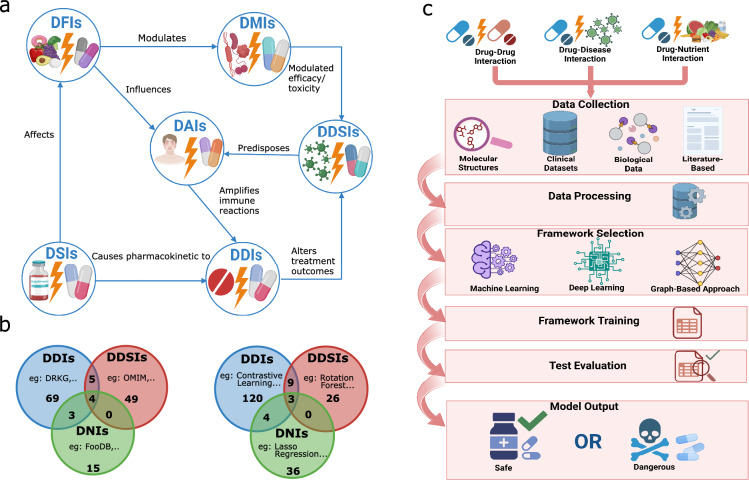
**a** Schematic representation of the six major categories of drug interactions and their interconnected relationships. **b** Venn diagram showing the distribution of dataset availability (left) and artificial intelligence model applications (right) across the three primary interaction types. **c** A flowchart of drug interaction prediction (drug–drug, drug–-disease, and drug–nutrient) utilizing various data sources, machine learning, deep learning, and graph-based methods for safety evaluation. Created with BioRender.com

Traditional laboratory methods for detecting drug interactions are slow and limited, making computational tools crucial for managing DDIs, DDSIs, and DNIs [2, 10–12]. DDIs are typically pharmacokinetic (PK)—affecting absorption, metabolism, or elimination—or pharmacodynamic (PD), involving shared targets that alter efficacy or toxicity [11–13]. DDSIs include disease-related exacerbation of drug effects and drug–allergy interactions (DAIs), where underlying conditions can trigger hypersensitivity [6, 14–16, 21]. DNIs, such as drug–food (DFIs), drug–supplement (DSIs), and drug–microbiome interactions (DMIs), influence drug behavior via enzyme modulation or gut physiology changes [17–20]. These interaction types often overlap; for instance, diet affects the microbiome, which makes DFIs modulate DMIs [9, 10], and DSIs can alter drug concentration and availability, thereby influencing DDIs [11]. DFIs can lead to DAIs when a shared substance, acting as both a food additive and a drug excipient, triggers a hypersensitivity reaction [12]. DMIs can modulate DDSIs, influencing drug efficacy and toxicity, while DDSIs may elevate the risk of DAIs in autoimmune or infectious diseases, and DDIs can worsen disease states that further impact DDSIs [13, 14]. These interconnections highlight the need for integrated, systems-level approaches to improve drug safety and therapeutic outcomes (Fig. 1a).

Artificial intelligence (AI) frameworks offer new opportunities for efficiently predicting and managing drug interactions [15]. This necessitates the creation of curated databases that cover diverse types of interactions such as DDIs, DDSIs, and DNIs as illustrated in Fig. 1b and enumerated in Supplementary Table S1. These datasets allowed the implementation of numerous AI models that can be classified as machine learning (ML, e.g. Random Forest), deep learning (DL; e.g. Convolutional Neural Networks or CNNs), and graph-based method (GBM; e.g. Graph Convolutional Networks or GCNs), as enumerated in Supplementary Table S1. ML-based models develop dynamic algorithms for data-driven decision-making [16], while DL-based models employ multiple layers of information processing to learn complex features and classify patterns automatically [17]. Conversely, GBM-based models can capture complex relationships between entities, a capability essential for understanding various aspects of drug interactions [18]. Despite the growing body of research on drug interactions, several gaps persist, including data imbalance, noisy sources, limited explainability, and underrepresentation of certain types of interactions (such as DNIs and DAIs). Current methods remain insufficient for capturing complex, interdependent relationships among DDIs, DDSIs, and DNIs, which limits their ability to predict adverse outcomes effectively.

This work represents the largest and most comprehensive exploration of AI's application in predicting and managing a wide range of multiple drug interactions. Given the inherent complexity and widespread impact of various drug interactions on patient safety, this review employs a systematic approach to consolidate and critically analyze existing research, methodologies, and AI-driven models across these diverse categories. By advancing these technologies, AI contributes to identifying novel drug targets and optimizing therapeutic strategies, which are critical in developing safer and more effective medical treatments (Fig. 1c).

## Methodology: Systematic approach for study selection

### PRISMA-guided search strategy

We adhered to the Preferred Reporting Items for Systematic Reviews and Meta-Analyses (PRISMA; [19]) checklist and flow diagram to ensure a rigorous and comprehensive literature review process (Supplementary Data S2). Six tailored search strategies were applied in PubMed for each interaction type—DDIs, DDSIs, DAIs, DFIs, DSIs, and DMIs—covering the duration between 2018 and 2025. For DSIs, the range was expanded from 2015, which was searched from 2015 to 2024, to include earlier key studies. Searches combined drug interaction terms (e.g., interaction, reactions, antagonism) with AI-related keywords (machine learning, deep learning, neural networks, natural language processing (NLP), generative models), focusing on titles and abstracts. For DDIs, we excluded terms linked to drug discovery, COVID, and cancer.

### Inclusion and exclusion criteria

We included studies that (1) used AI to predict drug interactions, (2) focused on multiple interaction types (e.g., DDIs, DDSIs, DAIs, DFIs, DSIs, DMIs), and (3) were peer-reviewed original research articles published between 2015 and 2024. We excluded studies that (1) were review articles, (2) had only abstracts, (3) were related to drug discovery, and (4) were specific to certain diseases like COVID-19 or cancer.

### Screening and study selection summary

**Fig. 2:**
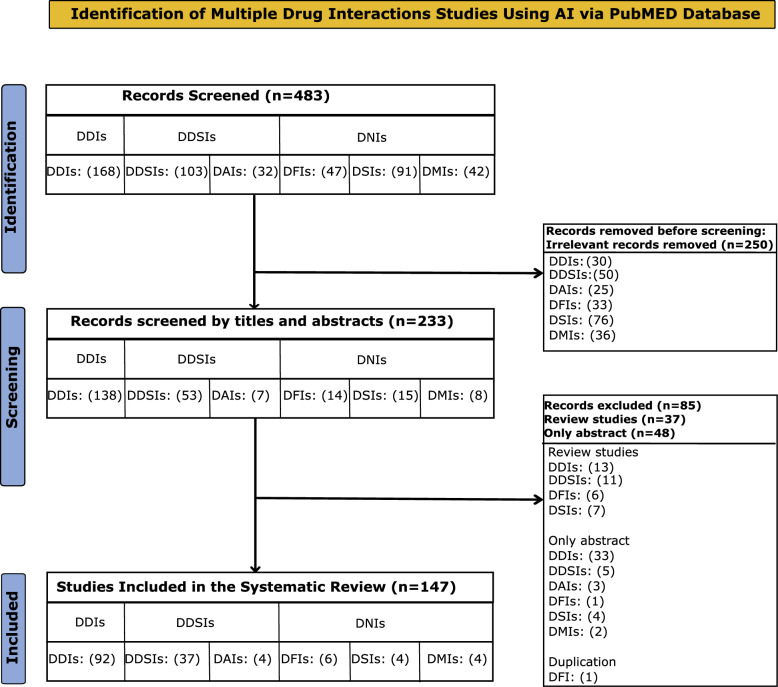
PRISMA chart of search results for every category of drug interaction.

After manual removal of irrelevant records, 233 studies remained for evaluation. We screened titles and abstracts, excluding 85 records lacking predictive models for drug interactions, including review articles and those without detailed methodologies. We manually reviewed full texts and extracted key information regarding study objectives, AI methods, interaction types, and evaluation metrics, without using standardized extraction forms. Ultimately, 147 studies detailing AI-based predictive modeling of drug interactions qualified for systematic review (Fig. 2).

### Scope and categorization of analysis

This review analyzes DDIs through AI methods such as ML, DL, transformer-based, and GBM. The discussion on DDSIs focuses on advancements in drug efficacy using ML, DL, and GBM techniques. DNIs were categorized into DFIs, DMIs, and DSIs, examining their impact on drug activity. The review also evaluates datasets used in drug interaction research, noting their strengths and limitations. Our review discusses the challenges in predicting drug interactions and therapeutic outcomes, concluding with broader field challenges like data heterogeneity and model interpretability (Supplementary Figure S1).

## Artificial intelligence techniques in drug–drug interactions prediction

**Fig. 3:**
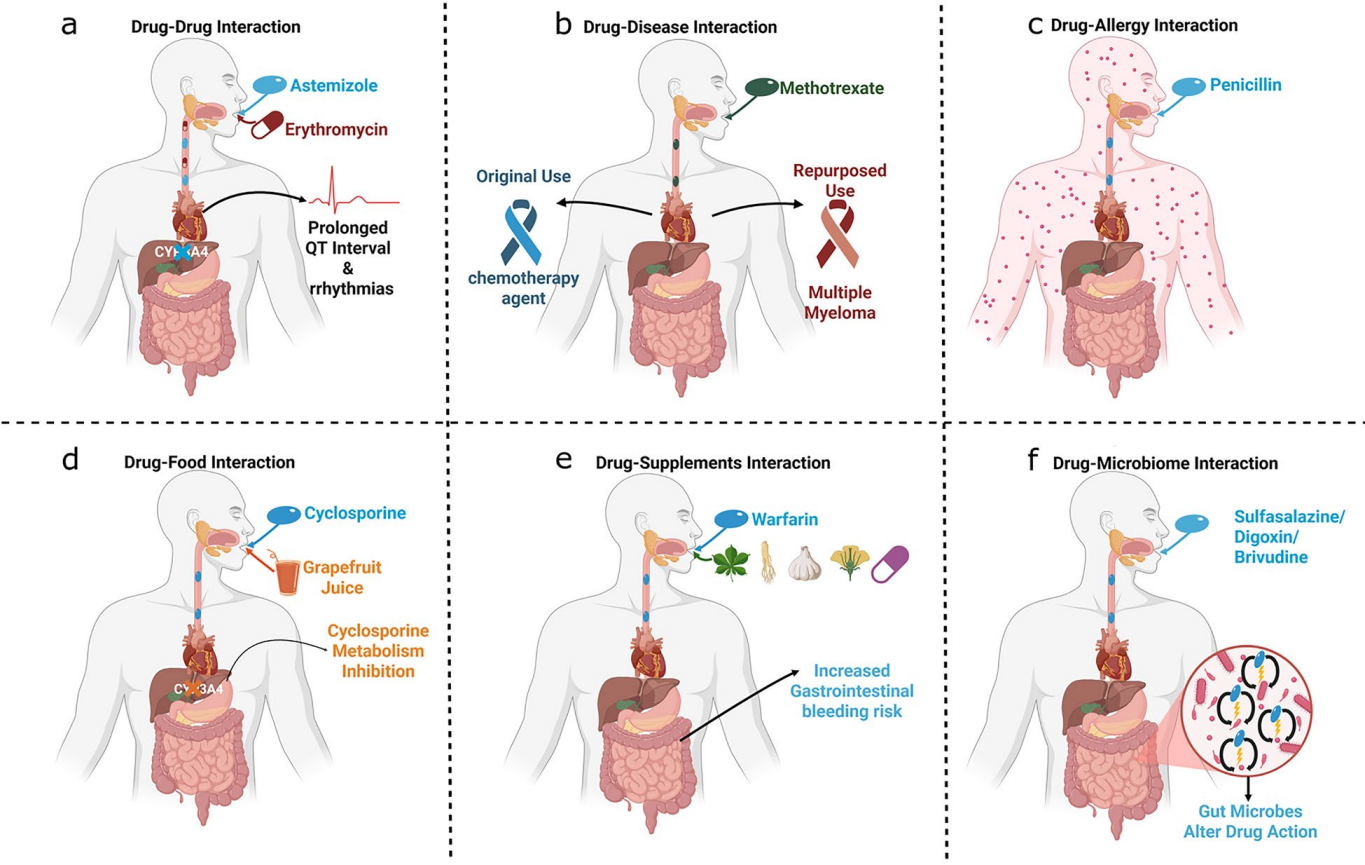
Representative case studies illustrating different types of drug interactions, including drug–drug, drug–disease, drug-allergy, drug–food, drug–supplement, and drug–microbiome interactions, each highlighting distinct mechanisms and clinical consequences.

**Fig. 4:**
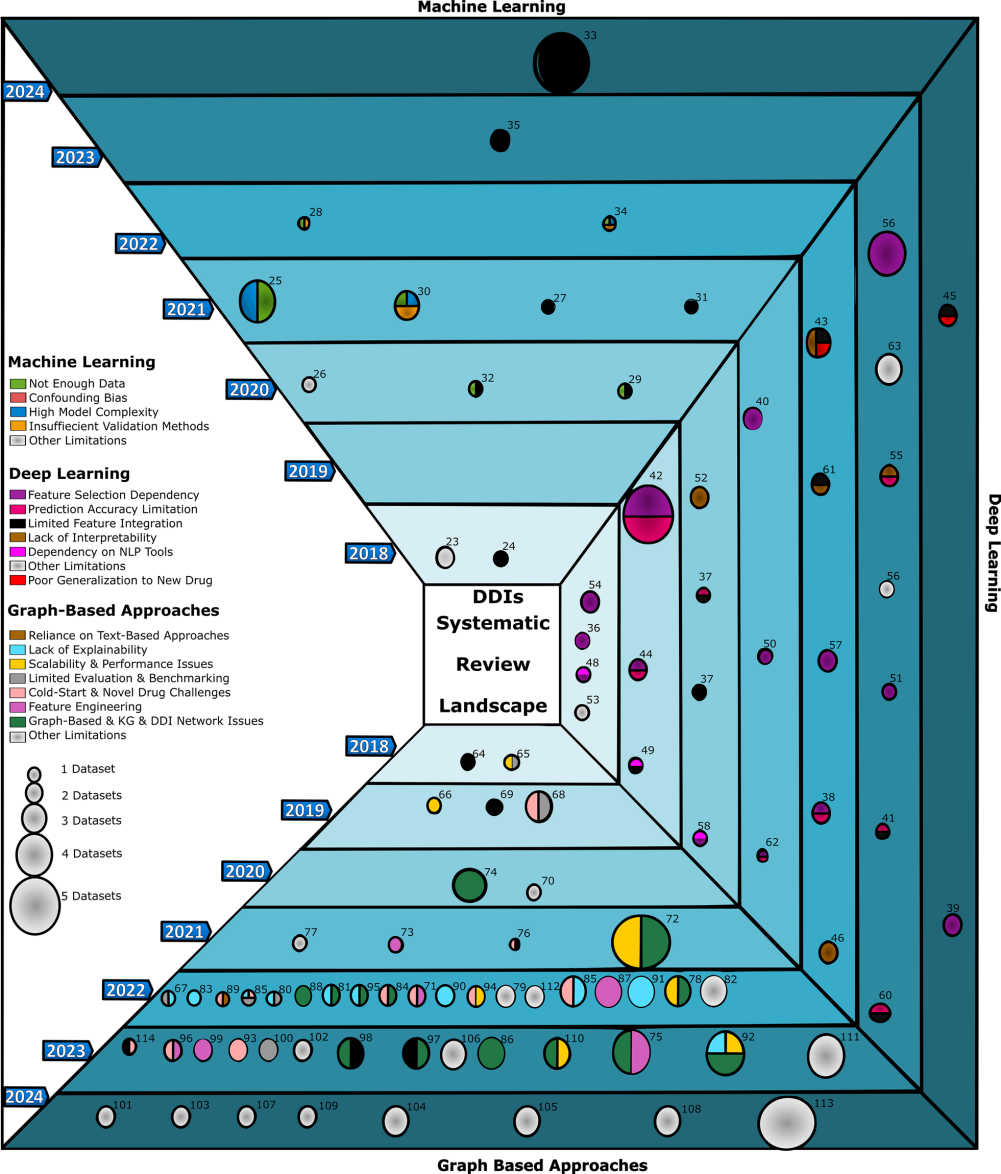
Chronological visualization (2018–2024) of AI approaches in DDIs research, showing the evolution of machine learning (top), deep learning (right), and graph-based method (bottom). Bubble size reflects dataset size; colors represent addressed challenges. Years progress from bottom to top within each method.

Several studies highlight how drug interactions, driven by biological mechanisms, can lead to harm or therapeutic benefit. For instance, the allergy drug astemizole was withdrawn after causing fatal heart rhythms when combined with erythromycin or grapefruit juice—both of which block the liver enzyme that metabolizes astemizole, leading to toxicity [20, 21]. Similarly, warfarin can become dangerously potent when combined with pain relievers like aspirin, as they compete for the same blood proteins. When aspirin displaces warfarin, it leads to excessive bleeding—a clear example of how drugs can interfere with each other’s distribution, as illustrated in Fig. 3a [22]. Over the years, researchers have developed extensive repositories to facilitate DDIs prediction, initially utilizing ML models and later advancing with an array of computational and AI approaches, including DL and GBM. As the field evolved, researchers adopted two different strategies regarding feature utilization in these models. Some researchers concentrated on using clinical and real-world observational data to predict DDIs. However, when comparing the performance of these models to those built by researchers who preferred to utilize pharmacoinformatic drug data, it was evident that the latter achieved significantly better results in DDIs prediction. This enhancement underscores the importance of integrating mechanistic insights into computational frameworks. Fig. 4 provides an overview of the methodological landscape, illustrating the evolution of ML, DL, and GBM over time, along with the key challenges addressed in each study.

### Machine learning approaches for DDIs predictions

ML has served as a key foundation to predict DDIs, Guiding researchers to better understand the problem and replacing the experimental validation, which was a time-consuming and expensive process. In early 2018, ML models such as k-Nearest Neighbors and support vector machine (SVM) were foundational—focusing on easy-to-access PK features, as these key features were more common and easier to obtain [23]. Early methods leaned on drug similarities, effectively paired in SVM kernels, which improved the ability to capture and mimic interactions between drug pairs [24]. Yet, integrating heterogeneous data proved difficult. To overcome this limitation, researchers utilized a simpler alternative using gene similarity alone in a regularized logistic regression model, enhancing both interpretability and biological relevance [25].

As the risks of DDIs increased, researchers gave more attention to high-risk DDIs using adverse event reports. Liu et al. tackled data scarcity with semi-supervised learning using autoencoders and weighted SVM [26]. Further advancements were made by extracting DDIs-related information from scientific literature with random negative sampling [27]. Electronic health records (EHRs) enriched pharmacovigilance by linking drug histories with adverse outcomes [28]. Some researchers worked beyond their initial models and utilized Bayesian confidence-propagating neural networks and ranking algorithms to confirm new signals [3]. To solve the cold-start problem to predict DDIs for new drugs, models like three-step kernel ridge regression achieved high performance as shown by the Area Under the Receiver Operating Characteristic Curve [29]. Some models were able to predict the quantitative predictions of the AUC) ratio in DDIs [20].

Distinguishing true DDIs from coincidental ones remains a major challenge. Sanjoy Dey et al*.*’s approach used data mining for initial filtering and employs logistic regression within causal interaction models to adjust for confounders, reducing false positives [30], while Wang et al*.* improved detection using a propensity score-adjusted mixture model, which applied logistic regression with propensity scores to improve DDIs detection [31]. Furthermore, other AI frameworks enhanced detection of DDIs by refining data sensitivity, clarifying causal interactions, and providing actionable outputs for clinical use, thereby advancing pharmacovigilance and the predictive accuracy of drug safety analyses [32]. Additional approaches used ensemble methods like Random Forest and AdaBoost to predict the high risk of corrected QT interval prolongation in the electrocardiogram caused by DDIs [33].

### Deep learning innovations in DDIs analysis

Although ML has widely been used for the prediction of DDIs, issues like overfitting and interpretability remain; consequently, researchers have turned to DL approaches to address these challenges. In response to these challenges, the DL approach offers the promise of interpretability as well as outperforming the ML approaches. For instance, Deng et al*.* were among the first to use deep neural networks (DNNs) with semantic analysis to classify 65 event types of DDIs, offering much deeper annotations [34], though overfitting persisted [34–36]. To mitigate this, CNNs were adopted for feature learning [35], and contrastive learning was further helped by pretraining on large molecular datasets to reduce overfitting by learning robust, transferable features from a diverse dataset before fine-tuning on a smaller labeled dataset [37]. This transition enabled researchers to move beyond binary interaction predictions and focus on specific events, such as synergistic or antagonistic effects [35].

Although most DL studies relied on molecular structure features, one approach stands out by combining them with external knowledge graphs (KGs) to learn multi-modal representations, boosting performance across tasks [38]. Yet, such methods still rely heavily on labeled datasets of DDIs—resources that are both costly and time-consuming to generate—as illustrated by Yang et al., who implemented a CNN using a Siamese network architecture [39]. To address this, Kpanou’s team employed self-supervised pre-training on large unlabeled datasets using contrastive learning [37]. Still, performance on novel compounds was suboptimal. Early neural network-based approaches for unknown DDIs showed promise, which was very encouraging as an initial step [40]. Several studies incorporated gene expression, gene ontology, and target gene similarity profiles using multi-layer neural networks with gating mechanisms or attention-based gating mechanisms to enhance interpretability [41, 42]. Further advancements included CNN-derived sequence features with mixed attention and substructure features via the substructure graph feature encoder [43]. Similarly, a study used a 3-D tensor of substructure–substructure–interaction triplets to predict DDIs, enabling both direct and inductive predictions with high interpretability [44].

Another method involves extracting DDIs from biomedical literature, which is crucial due to the rapid growth of publications containing up-to-date information on DDIs [45]. DL methods, including BiLSTM (Bidirectional Long Short-Term Memory) with attention mechanisms [46–50], CNNs with pooling [51], network-based analysis frameworks, and Recursive Neural Networks with tree Long Short-Term Memory [52–54], have automated this process. However, some approaches struggle with words that have multiple meanings that could led to contradictory interpretations. To address this, the utilization of the Bidirectional Encoder Representations from Transformers for Biomedical Text Mining (BioBERT) and CNNs with attention enabled better representation and handling of ambiguity [45, 55]. Imbalanced data, which can lead to overfitting, was tackled using hybrid networks combining the Bidirectional Gated Recurrent Unit model and CNNs, as well as Gaussian noise-based methods to improve model robustness and active learning with targeted sampling [56–58]. Another study solved this issue using focal loss, multi-source drug fusion, multi-source feature fusion and the transformer self-attention mechanism, which helped achieve more robust prediction, particularly for the cold-start issue [59].

Error propagation in pipeline models was reduced through modular BioBERT-based pipelines with rule-based data refinement [60]. The directionality of DDIs, crucial for PK insights, was tackled by a fine-tuned BioBERT model that classified sentences and identified object-precipitant roles for actionable insights [61]. However, challenges remain, including overfitting, high computational demands, and dependence on large, often scarce labeled datasets. Moreover, early DL methods relying on text-based techniques and single similarity measures have struggled to capture structural and neighborhood-level information.

### Graph-based method for DDIs prediction

GBM in the prediction of DDIs has advanced by addressing ML and DL challenges through structured data, unified kernels, convolutional schemes, directed graph attention networks, and artificial neural networks to improve interpretability [62–65]. Key challenges—such as generalization to new drugs, biases in prediction, and scalability in high-order DDIs—were tackled through disjoint cross-validation schemes, heterogeneous KGs, and ensemble classifiers [63, 66]. Further innovation was achieved through GCNs by enabling detailed substructure-specific analysis and adaptive sampling, which improved accuracy in real-world settings [67, 68]. Additionally, using SMILES-based structural features along with topological features and aggregating diverse data sources provided a better solution using Graph Neural Networks (GNNs) [69]. To manage noise, sparsity, and complexity, models integrated high-quality drug KGs with translation-based embeddings, autoencoders, and GCNs [70, 71]. Adversarial autoencoders using Wasserstein distances generated more plausible negative samples [72], while neural factorization machines effectively modeled higher-order feature interactions [73]. Graph attention mechanisms—like GATs and deep attention neural networks—also performed well by explicitly modeling intra-drug interactions and enhancing interpretability via co-attention [74, 75].

In 2022, GBM in DDIs saw transformative advancements, with breakthroughs in feature integration and generalization tackled by multi-type feature fusion and GNNs enabling integration of molecular structures, interaction data, and topological relationships [76, 77]. Some models encoded 2D drug structures with spatial information using self-attention and Siamese networks, addressing the limitations of available data [78]. Meanwhile, the lack of 3D structural representation was confronted Head-on by several studies, which introduced 3D molecular graphs, named SciBERT, and position embeddings, deepening molecular feature interpretability and bridging domain-specific gaps [79, 80]. Attention mechanisms like dual attention-aware networks and KGs embeddings took a central role in several studies, where the focus shifted to enhancing semantic relationship modeling and higher-order connectivity [81–84].

By using multi-dimensional representations, these approaches significantly boosted prediction accuracy and adaptability. Moreover, KGs can effectively make uncertain decisions by combining them into a three-way decision-based model to enhance the prediction of DDIs [85]. On the other hand, other researchers prioritized substructure-specific interactions, using co-attention mechanisms and layers of GNNs tailored for molecular subcomponents [86, 87]. This innovation allowed accurate predictions for unseen drugs while bypassing the reliance on predefined features. To address scalability and computational inefficiencies, researchers advanced the use of scalable network embedding methods, such as random walk and GNNs, by introducing the solution of hypergraph neural networks [88–90]. Using sparse representations, these models modeled high-order relationships among drugs and side effects, excelling in multi-type DDIs prediction scenarios. Cold-start challenges were also innovatively tackled by a lookup adjacency matrix factorization (MF) with propagation using a similarity-based lookup mechanism [91]. Others handled it by embedding drug attributes and employing generalized mapping functions, ensuring robust predictions across real-world datasets, which achieved higher performance [92, 93].

By 2024, a shift towards integrating diverse perspectives and data sources drove innovation. Dual-view learning frameworks like the DSN–DDI model [94] addressed both intra-drug and inter-drug representations, while the HetDDI model used heterogeneous GNNs to capture diverse KGs relationships [95]. Studies focused on multimodal fusion, combining molecular graphs, biochemical features, and KGs to reduce redundancy and prioritize impactful interactions [96, 97]. Relation-aware models tackled asymmetric DDIs through directed relation embeddings and bidirectional refinement, enabling precise role-specific insights [98, 99]. For unseen drug predictions, the AutoDDI model [100] can efficiently design the architecture of GNNs for the prediction of DDIs without manual intervention, while another study [101] introduced adaptive mechanisms to refine substructure-level features and handle noisy molecular data. The model KGRLFF, on the other hand, addressed scalability by extending sampling to higher-order relationships with multi-entity designs [102].

The drive for interpretability was evident in studies that used visualization tools and knowledge subgraphs to explain predictions and foster trust in AI-driven models [22, 103]. There is a limitation related to the dependency on comprehensive drug information, like the high-quality drug interaction networks, and the dependency on large training data, which can be solved by using fingerprints only, combining fingerprints with the Graph Sample and Aggregate model, and employing unsupervised contrastive learning [104–106]. These efforts bridged computational advancements with clinical applicability, culminating in robust, scalable and interpretable solutions for DDIs prediction. Recent papers address the limitation of binary prediction of DDIs by adopting advanced approaches. One model predicts multi-typed DDIs through multi-channel feature fusion, combining drug structure, label, and KGs features with GRU and State Encoder [107]. Another integrates molecular structure with semantic features from a biomedical KGs, optimized with a multi-head attention mechanism [108]. GBM has been effective in extracting DDIs information. These methods use multi-aspect graphs and multi-scale embeddings from KGs, SMILES sequences and molecular structures might be optimized with self-attention mechanisms [109, 110]. Additionally, frameworks combining multi-scale drug embeddings achieved an improved AUC [111].

### Artificial intelligence techniques in drug–disease interactions prediction

DDSIs occur when a medication prescribed for one condition negatively impacts another coexisting condition and its treatment [4]. Studies estimate that up to 30% of older adults with multiple chronic conditions may be affected by such interactions [112]. While harmful, DDSIs can also present opportunities for drug repurposing, which involves reusing a medication to treat various diseases [113]. It is essential to predict DDSIs for preventing the side effects and for repurposing drugs to offer safer, more cost-effective solutions with shorter development times [112]. Beyond DDSIs, DAIs have also been identified as a critical concern. Allergies, as chronic inflammatory disorders, can trigger harmful or adverse reactions when certain medications are administered, making it essential to incorporate them into predictive models [114, 115].

Several case studies demonstrate how AI-driven models have successfully uncovered new therapeutic uses for existing drugs by predicting DDSIs with clinical relevance. Using the PUON model trained on known drug–disease pairs, researchers found that doxorubicin, originally used for certain cancers, was predicted to also be effective against Kaposi sarcoma and esophageal cancer, both of which were validated through the Comparative Toxicogenomics Database. Similarly, methotrexate, a chemotherapy agent, was identified as a candidate for multiple myeloma, and risperidone, primarily used for psychiatric disorders, was linked to new disease associations supported by biological databases [114]. In the context of neurological disorders, a random walk and supervised learning model revealed that phenylhexol, not previously indicated for Parkinson’s disease, interacts with disease-related proteins like α-synuclein and tau, suggesting a new mechanism for treatment that had not yet been reported in clinical databases [115]. These findings offer real-world, biologically grounded examples of how AI can identify new, plausible disease targets for known drugs, advancing the goals of safe drug repositioning (Fig. 3b).

### Machine learning for DDSIs prediction

ML is the dominant approach in DDSIs research, effectively addressing the high cost and time constraints of wet lab experiments. Initially, researchers relied on MF with drug feature-based and disease-semantic similarities, though this approach introduced noise [116]. To improve accuracy, heterogeneous networks were introduced, integrating multiple similarity measures [117]. These models also eliminated the need for negative training sets through semi-supervised models or drug–disease–gene tripartite networks, prioritizing credible DDSIs [117, 118]. One example is the model entitled RepCOOL, which integrates heterogeneous biological networks and employs a random forest classifier to enhance DDSIs predictions, addressing data sparsity and network-based modeling challenges [119]. Nevertheless, errors in heterogeneous networks arose due to limitations in known DDSIs, steering researchers towards incorporating Gaussian interaction profile (GIP) kernels and L2,1-norm regularization to reduce interference and overfitting [120].

Despite these improvements, existing methods still struggle with integrating high-dimensional drug features, managing noise and data loss, and predicting associations in heterogeneous datasets due to inefficient feature integration. The non-negative matrix factorization (NMF) method effectively addressed these issues by projecting features into low-dimensional spaces, preserving feature diversity, and optimizing similarity fusion [121–123]. One study further refined NMF by dynamically adding bilinear MF to ensure biologically meaningful and accurate predictions [122]. Yet, these solutions still rely on the binary prediction of DDSIs [124], overlooking multiple types (e.g., therapeutic vs. non-therapeutic) and limiting accuracy [125]. A study addressed this limitation through multi-task learning and matrix tri-factorization to integrate diverse association types and prior knowledge, while the work of Lu et al*.* reported using a Restricted Boltzmann Machine for better performance [124, 125]. Another study improved predictions using a supervised Random Walk with Restart approach, integrating multiple databases and selecting a gene set as the starting point, allowing known DDSIs to guide the learning process [115]. To tackle the challenges of insufficient known DDSIs and ineffective data utilization, the work of Wang et al*.* applied graph-regularized NMF, incorporating weighted nearest neighbor reconstruction to infer interaction profiles for novel drugs and diseases, resulting in high AUC performance and significantly reducing data sparsity [126].

The use of gene expression data with ML has emerged as a powerful strategy to enhance DDSIs predictions by integrating disease-related gene activity with drug–treated expression profiles [127, 128]. Another method applies tensor decomposition with neural networks to model complex drug–gene–disease associations [128]. Additionally, a systems biology approach that integrates gene expression signatures with signaling pathways has been used to construct a drug–disease network, enhancing drug–repurposing predictions [129]. These methods offer a significant improvement by capturing both linear and non-linear interactions, thus improving drug repositioning and treatment prediction.

Another aspect that plays a crucial role in DDSIs for identifying new drug targets is the protein interaction analysis [130, 131]. One study developed a ML model to predict protein–protein interaction hotspots, identifying potential drug candidates for diseases linked to EphB2 mutations [130]. Another study complemented this by using a random forest model to integrate multiple protein similarities, improving DDSIs predictions with high accuracy [131]. Both studies highlight the role of computational methods in using protein interactions for drug discovery and repositioning. A unique approach proposed using medication data to identify frequently contraindicated diagnoses, applying expert-based and machine-learning algorithms to enhance medication safety in community pharmacies and clinical practice [132].

### Deep learning models for DDSIs associations

While DL has been increasingly explored for DDSIs prediction, it remains less widely adopted compared to traditional approaches. One of the most widely used approaches with DL models for DDSIs prediction is the GIP kernel to enhance feature representation [133–136]. GIP is sometimes coupled with auto-encoders to reduce noise and improve feature extraction or with deep-gated recurrent unit models to overcome limited feature representation [133, 134]. Among these efforts, DenseCNN—a model that combines GIP with a convolutional block attention module—demonstrated one of the strongest performances [136]. For greater stability and robustness, sparse autoencoders have also been combined with GIP and rotation forests [135].

Beyond GIP-based methods, DL has addressed DDSIs prediction challenges by refining feature extraction and model robustness [137, 138]. Early efforts, such as similarity network fusion with neural networks, improved predictive performance but struggled to integrate diverse data types effectively [137]. Later approaches constructed heterogeneous networks, extracted topological features, and applied DNNs to mitigate overfitting [138]. However, these methods relied on shallow models that struggled to capture complex, nonlinear DDSIs. A notable advancement is HeteroDualNet, a model with dual CNNs model that integrates intra-drug similarity, intra-disease similarity, and DDSIs into heterogeneous layers [139]. This dual-branch architecture improves predictions by learning from both direct and neighboring drug–disease relationships. Other CNNs-based methods further optimized DDSIs predictions, like a Sigmoid Kernel-based CNNs that integrated multiple similarity measures to extract meaningful features while addressing data sparsity and noise [140]. Not only that, but also CNNs were able to improve generalization by incorporating molecular structures and clinical symptoms while overcoming shallow model limitations by combining with BiLSTM to capture both local and sequential features [141, 142]. Despite the success of DL in addressing key limitations, remaining challenges and growing interest in the prediction of DDSIs are driving researchers toward GBM as the next step forward.

### Graph-based method for DDSIs prediction

The GBM in DDSIs is not as well-established as ML or DL methods. This can be due to the novelty of using GCNs in DDSIs research, which began in 2021, marking the start of this methodology [143–145]. Early studies used GCNs with attention mechanisms to integrate heterogeneous networks, improving feature propagation and scalability through node similarity-based convolution matrices for link prediction [143, 144]. Further progress came with graph convolutional auto-encoders, which integrated multi-source data, node attributes, and raw features for higher prediction accuracy [145]. Recently, experts adopted GCNs to address the limitations of existing methods in modeling complex, multi-dimensional relationships [146–148]. To overcome these challenges, GCNs-based frameworks incorporating auto-encoders, attention mechanisms, and link prediction were developed to integrate diverse data sources, construct heterogeneous graph structures with event nodes, and model drug-target-disease relationships [146, 147].

Beyond standard GCNs, newer methods have introduced more advanced graph frameworks to better capture heterogeneous interactions and global connectivity. KGs-based models integrated genotypic and phenotypic data to enhance drug–disease inference [149], while GNN-based models like GDRnet used multi-layer heterogeneous graphs to model complex interactions [150]. REDDA used heterogenous GNNs and attention mechanisms for improved embedding learning and interpretability [151]. Moreover, DTD-GNN introduced heterogeneous graph structures with event nodes to model drug-target-disease relationships, combining GCNs and GATs for enhanced feature representation and link prediction, suggesting that further trials could yield improved results [147]. Moreover, DT2Vec+ employed heterogeneous graph mining by integrating drug–drug, protein–protein, and drug/protein–disease associations into low-dimensional vectors to predict drug–target interactions and their types [152]. The field of DDSIs prediction has seen significant progress, with a wide array of approaches being explored. Despite the promising potential of GBM, it remains underexplored. Advanced graph frameworks offer the potential to model complex relationships more effectively, and their full capabilities in DDSIs prediction have yet to be fully realized. Additionally, further exploration of literature-based approaches, primarily through transformers and similar models, could provide access to more datasets, enhancing the scope and accuracy of DDSIs prediction.

### Artificial intelligence techniques in drug–allergy interactions

Accurate documentation of DAIs is vital for safety, yet gaps in EHRs persist. In one study, 7% of allergy records remained uncorrected after negative drug challenge tests, risking treatment errors and adverse outcomes, as visualized in Fig. 3c. While DDSIs get more attention, DAIs—harmful immune reactions caused by medications—are also critical, as they can lead to chronic inflammation or even life-threatening effects [5, 153]. To improve the detection of DAIs, several studies applied advanced NLP and ML techniques. At Beijing Children's Hospital, a study found that the best-performing approach to automatically identify and classify rare, severe drug hypersensitivity reaction cases involved key sentence selection combined with a domain-specific transformer like ClinicalBERT, followed by an ML classifier [154]. Other tools like MTERMS extracted free-text and medication data, mapped them to SNOMED-CT, and standardized allergy documentation [155]. Another study employed ML models that integrated 22 structured data features with key symptoms identified in clinical notes through NLP to detect allergic transfusion reactions [156]. The work of Lo et al*.* used a rule-based NLP system to detect mismatches between drug challenge test results and allergy lists, enabling real-time alerts and improving patient safety [157].

## Artificial intelligence techniques in drug–nutrient interactions prediction

DNIs arise from physical, chemical, physiological, or pathophysiological relationships between a drug and a nutrient [158], potentially increasing nutritional risk by causing micronutrient deficiencies, which can reduce a drug's effectiveness and amplify its side effects [159]. The widespread use of polypharmacy, especially in older adults with comorbidities (affecting 40–55%), is a key driver of DNIs occurrence [160]. Recent advances in AI and computational models offer powerful tools to predict and manage DNIs and related interactions like DFI, DMI, and DSIs.

### Drug–food interactions modeling with artificial intelligence

Dietary components can alter drug efficacy and safety by affecting metabolism, absorption, and bioavailability. As shown in Fig. 3d, certain food compounds like levoglutamide, l-glutamic acid, and spermidine (from oats and beetroot) reduce concentrations of up to 30 hypertension drugs [53]. Given the complexity of Such interactions, AI has played a vital role in improving DFIs. Research began in 2018 with DNN-based frameworks using SMILES and drug names to predict the pharmacological effects of 256 foods and the bioactivities of 149 food constituents, achieving notably high accuracy [36]. Subsequent approaches incorporated ML techniques, such as XGBoost for the extraction of DFIs and Random Forest, SVM, and KNN for analyzing interactions between food additives and drug excipients, particularly their ability to block enzymes and transporters [161]. Other ML studies applied XGBoost to extract features from drug–food compound pairs [162]. More recently, GBM such as FDMine and DFinder have used graph mining and GCNs for top-tier performance [163, 164]. NLP and text mining techniques have facilitated the development of datasets like FooDrugs and annotated corpora for DFIs from scientific literature [165, 166]. As the field evolves, integrating multimodal data sources and enhancing model interpretability will be crucial for improving the prediction of DFIs.

### Drug–supplement interactions using artificial intelligence

Unmonitored DSIs can pose serious risks. Clinical data linked warfarin with supplements like garlic, ginkgo, ginseng, St. John’s Wort, and Vitamin E—raising the risk of adverse events such as gastrointestinal bleeding (Fig. 3e) [167]. Three studies employed NLP to mine diverse data sources and identify novel DSIs of potential clinical relevance [167–169]. A model predicted the interactions between warfarin and dietary supplements using NLP to extract information from clinical notes, then used statistical models like Cox proportional hazards to identify the risk of adverse events [167]. Zhang et al*.* expanded on this by mining biomedical literature using lasso regression, SemRep and semantic pathways like supplement-gene and gene drug [168]. SuppKG, one of the specialized KGs for DSIs, was implemented by the PubMedBERT model and outperformed prior methods by generating novel DSIs hypotheses, providing a more comprehensive tool for DSIs prediction and analysis [169]. Building on this, recent work has explored Drug–Herb Interactions (DHIs), which affect drug metabolism similarly. For instance, elevated levels of the tacrolimus drug were reported in a renal transplant patient after consuming Fructus Citrus maxima [179]. To support such clinical findings, a GBM was developed using cardiovascular drugs and herbs, with *node2vec* achieving top performance.

### Drug–microbiome interactions using artificial intelligence

Growing interest in DMIs has led to advanced methods and deeper clinical insights. Experimental evidence, illustrated in Fig. 3f, shows that microbiota modulate drug metabolism—for instance, activating sulfasalazine, inactivating digoxin, and converting brivudine into toxic metabolites, with these effects linked to individual microbiome variation [170, 171]. One study curated 455 cases of DMIs and used a tuned, extremely randomised trees classifier to predict a drug’s susceptibility to microbial metabolism [171]. Another combined drug chemical properties with microbial genomic features, using random forest to predict effects of individual drugs on microbial growth and broader shifts in microbial communities [172]. Recent efforts now focus on how drugs alter the microbiome itself [173, 174]. Thirteen ML models were developed to evaluate drug effects on gut bacterial strains, which led to the creation of predictive tools that assess potential drug-induced dysbiosis and associated health risks [173]. Another study utilized a dynamic artificial gastrointestinal tract model called ABIOME with Multivariate Adaptive Regression Splines ML model to analyze interactions between probiotics, predicting synergistic effects on metabolite production [174].

## Datasets for drug interaction prediction

A diverse range of datasets has shaped advancements in drug interaction prediction, spanning various interaction types such as DDIs, DDSIs, and DNIs, including those involving food, herbs, the microbiome, and supplements. Over time, several repositories have emerged as foundational resources, offering structured insights that have driven the development of AI-driven models. As predictive models continue to evolve, access to well-curated and diverse datasets becomes increasingly essential for uncovering complex interaction patterns and enhancing predictive capabilities.

### DDIs and DDSIs datasets

The exploration of both DDIs and DDSIs relies on a diverse set of databases, each offering crucial insights into various aspects of drug behavior and their relationships with diseases. These include DrugBank, TWOSIDES, KEGG, SIDER, PubChem, and OMIM, each playing a distinct and significant role in different areas of research [175]. DrugBank, a comprehensive repository, stands at the center of both DDIs and DDSIs research, with over 17,000 entries detailing small molecules, biologics, and experimental drugs and their targets [175]. TWOSIDES complements this by capturing side effects resulting from multidrug use, including 1317 side effects on 645 drugs across 63,473 drug pairs [176]. Similarly, KEGG plays a dual role, mapping drug pathways for DDIs while integrating genomic and systemic data to reveal DDSIs [177]. SIDER and PubChem support DDIs and DDSIs studies by linking drug mechanisms to side effects and providing chemical structure data for side effect prediction and drug repurposing [178, 179]. For DDSIs specifically, databases like OMIM focus on human genes and genetic disorders, providing gene-disease associations that are key for understanding phenotypic similarities and disease definitions [180].

Advanced DDIs data is available in resources Like DeepDDI, which offers a multi-class dataset of nearly 200,000 drug pairs, while DRKG expands the scope by integrating drug, gene, and protein relationships using the frameworks of KGs [36, 181]. OFFSIDES and CTD fulfill more targeted roles: OFFSIDES focuses on identifying off-label side effects not captured in clinical trials, and CTD facilitates DDSIs by curating chemical–gene–disease associations [182, 183]. Building on DDSIs research, benchmark datasets such as Fdataset and Cdataset serve as gold standards [184, 185]. The Fdataset, with 1,933 DDSIs sourced from DrugBank and OMIM, offers verified predictions, while Cdataset extends this by integrating additional associations with drug similarity and phenotypic information [184, 185]. As for DAIs, studies typically use EHRs from private healthcare systems. For example, one dataset from Beijing Children's Hospital includes over 431,000 hospitalization records from 315,608 patients, covering diagnostic, medication, and laboratory data between 2012 and 2020 [154]. Another dataset from the Mass General Brigham system includes patient records with at least one active allergy entry [157]. Additionally, a transfusion dataset of 86,764 cases with 146 reported allergic reactions was used to evaluate transfusion-related adverse events [156].

### DNIs datasets

DSIs are supported by a range of data sources and specialized tools. PubMed provides curated abstracts that enable the extraction of semantic relationships connected to DSIs, facilitated by the custom NLP tool SemRepDS and the dietary supplement-specific database iDISK [169]. Additionally, SemMedDB further contributes to the research of DSIs with over 69 million semantic predications derived from 23.6 million MEDLINE citations [168]. Some studies also utilize EHRs, like the University of Minnesota’s repository, covering over 2 million patients, offering deeper clinical insights into DSIs [167]. For DFIs, data sources include comprehensive information on drug targets, food compounds, and nutrient content. Text mining tools, such as DFinder, extract DFIs from PubMed and DrugBank [164, 166], while tools like IIG and GRAS lists from the FDA complement this by converting data into bioactive compound structures [161]. FooDrugs also integrates data from DDICorpus, PubMed, and ClinicalTrials.gov data and applies transcriptomic profiling using Gene Expression Omnibus and Connectivity Map for further analysis [165].

**Fig. 5:**
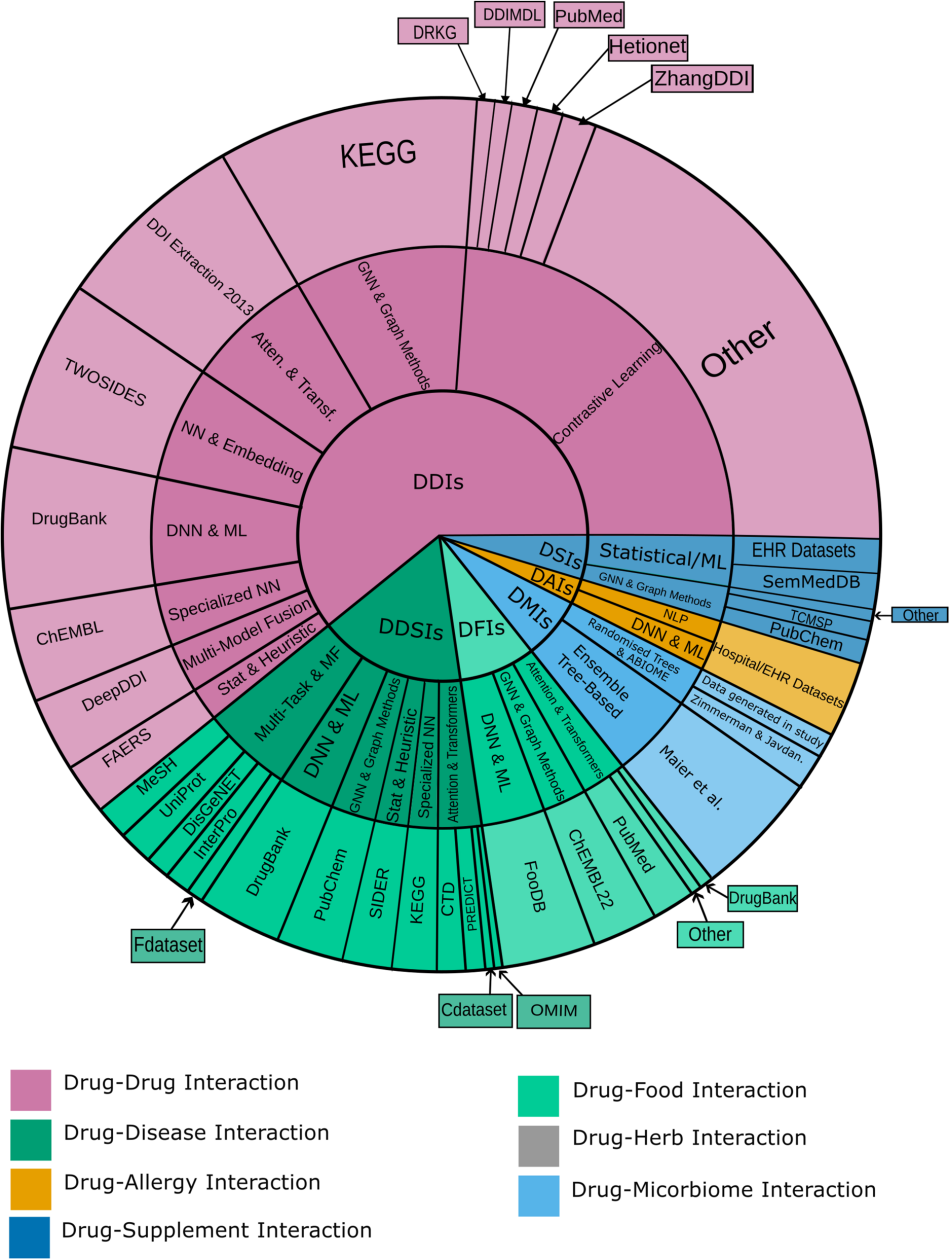
Sunburst plot of common datasets and AI models used across different drug interaction types, with sizes proportional to their frequency of occurrence in the literature.

Some studies developed datasets to examine DMI, linking gut bacterial species to drug metabolism. One study used genomic and metagenomic data to assess bacterial drug-metabolizing capacities, providing insights into DMIs [170]. Another dataset contained 41,519 DMIs and was derived from an in vitro screen of 40 microbial strains exposed to 1197 drugs. This dataset predicted interactions using 148 microbial genomic features, 92 drug features, and data from DrugBank and KEGG [172]. For DHIs, studies often rely on datasets related to cardiovascular disease herbs [186]. Researchers use DrugBank, DrugCentral, TCMID, ETCM, TCMSP, and PubChem databases to predict drug targets and assess pharmacokinetics [187]. The availability of datasets varies widely across drug interaction types, impacting model performance. DDIs are supported by rich, accessible datasets like DrugBank and ChEMBL, enabling advanced AI models; however, these datasets would benefit from more patient-level and longitudinal data to capture real-world variability and long-term interaction effects. Meanwhile, DNIs, including DFIs and DMIs, face limitations due to a lack of extensive public datasets. DDSIs and DAIs also require multi-source integration for comprehensive insights, though additional public, disease-specific data would enhance predictions. Expanding dataset availability and richness, particularly for DNIs, is crucial to advancing personalized medicine and supporting data-intensive AI models (Fig. 5).

## Discussion

**Fig. 6:**
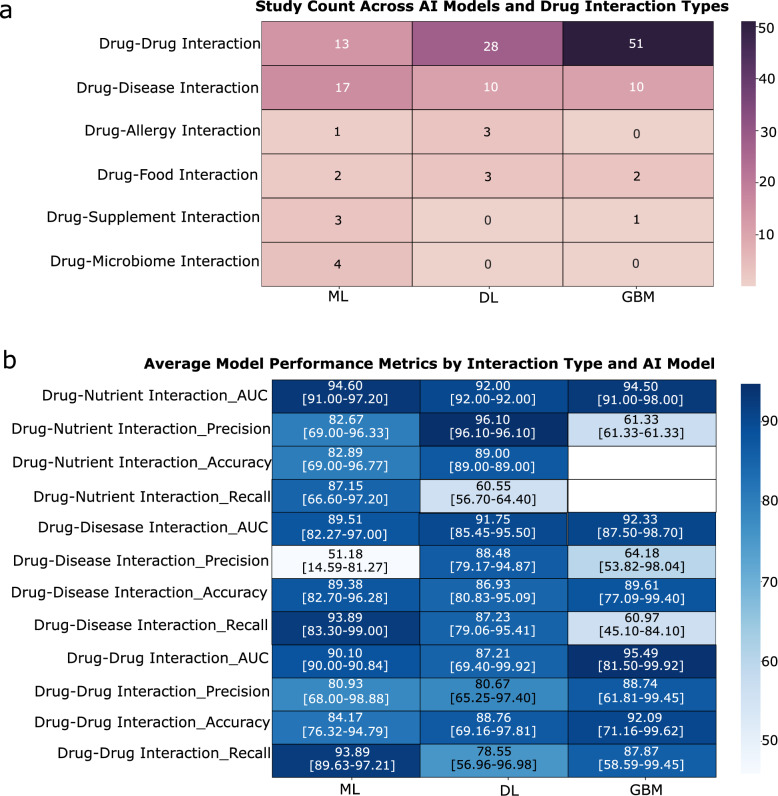
**a** Heatmap showing the number of studies investigating each drug interaction type using different AI model categories. **b** Heatmap displaying the average values of key performance metrics reported for each drug interaction type and AI model category.

The distribution of studies across AI methodologies and interaction types reveals clear patterns and notable gaps. Drug–drug interactions (DDIs) dominate the field, comprising about 55% of all studies, with graph-based method (GBM) accounting for over half, reflecting their alignment with the networked nature of DDIs data. Deep learning (DL) is also prominent, especially in DDIs and drug–disease interactions (DDSIs) studies, representing roughly 20% and 10%, respectively. Machine learning (ML) shows broader applicability across interaction types. In contrast, drug–allergy (DAIs), drug–food (DFIs), drug–microbiome (DMIs), and drug–supplement (DSIs) interactions are underexplored, each making up less than 5% of studies (Fig. 6a).

To assess model performance in predicting drug interactions, key classification metrics are applied. Accuracy reflects the proportion of correct predictions, and recall compares the number of correctly predicted positive examples to the number of positive examples, including those incorrectly predicted as negative [188]. Precision is the ratio of correctly predicted positive examples to all examples predicted as positives, and the F1-score is the harmonic mean of precision and recall [188]. GBM consistently delivers the highest Area under the curve (AUC) and strong precision and accuracy, making it the most effective in the tasks of DDIs. DL methods offer competitive recall and precision, particularly in the studies of DDSIs and drug-nutrient interactions (DNIs). The lower precision in some GBM results likely reflects data limitations rather than model flaws. Overall, GBM shows strong and often underrecognized performance across interaction types, reinforcing its value for future research (Fig. 6b).

**Fig. 7:**
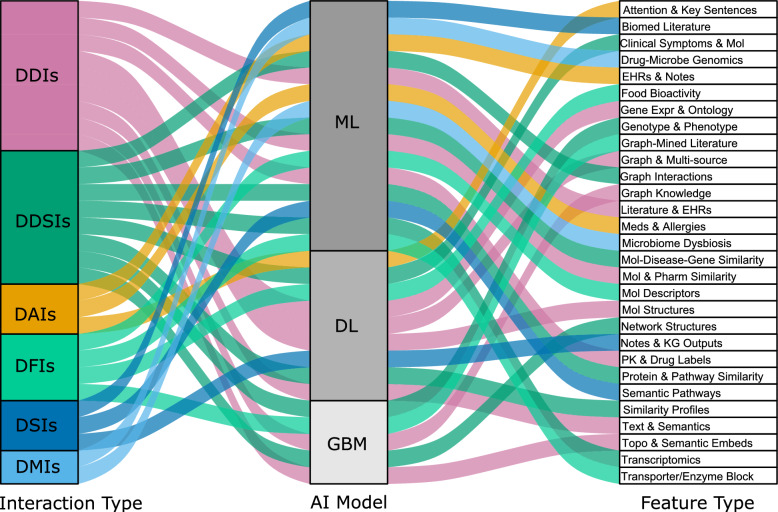
Alluvial plot illustrating the relationships between drug interaction types, AI model types, and utilized feature categories in predictive modeling

To better understand why GBM excels in DDIs prediction, it is important to examine the AI approaches driving their success. Recent advances have focused on integrating structured graph-based knowledge with deep learning, enabling models to capture complex semantic and relational drug patterns [73]. In parallel, multimodal feature fusion (Fig. 7)—combining molecular structures, biochemical properties, and interaction data as topological and semantic embeddings—has significantly improved accuracy and robustness [76, 96]. Dual-view learning, which captures both local substructures and global representations, has demonstrated strong generalization in inductive settings [94], while relation-aware refinement and consistency training have enhanced model reliability across datasets [98].

Building on their success in DDIs prediction, DL and GBM have also demonstrated strong performance in DDSIs prediction. As illustrated in Fig. 7, DL models stand out for their ability to learn from heterogeneous network structures, using Gaussian Interaction Profiles with autoencoders or gated recurrent units to stabilize and enrich feature learning [134, 135]. Performance improves further with attention mechanisms and dual-branch convolutional networks that capture both local and sequential signals [136, 139]. Meanwhile, GBM addresses scalability and interpretability by modeling multi-source biological relations through graph convolutions and attention layers [143, 151]. Advanced models further encode genotypic and phenotypic data, integrating drug-target-disease associations via event nodes and layered interactions [145, 147].

Addressing DAIs is critical, as they can be life-threatening. Enhancing prediction accuracy through AI is essential for patient safety [5, 153]. Likewise, improving the prediction of DFIs will rely on integrating multimodal data—clinical, genomic, and environmental [161]. The rise of GBM and natural language processing (NLP)-enhanced datasets promises more accurate, scalable, and clinically relevant predictions [165, 166]. Incorporating microbiome data could further revolutionize AI-driven models by uncovering drug-induced dysbiosis and enabling more personalized therapies [170, 189]. These advances pave the way for precision medicine and transformative improvements in patient care (Fig. 7, Supplementary Table S1).

### Challenges and opportunities

AI-driven drug interaction prediction faces several key challenges: (1) the lack of well-validated negative examples, leading to imbalanced datasets and complicating evaluations [27, 72]; (2) the integration of diverse data sources (e.g., molecular structures, literature, and pharmacoinformatic and clinical records), which is hindered by noise and incompleteness; (3) limited explainability and trustworthiness of predictive models, particularly in clinical settings where transparency and justifiability are essential; (4) the difficulty that traditional models face in handling unstructured and complex data, such as clinical notes, research papers, and drug labels [36]; (5) resource constraints that limit scalability; and (6) underrepresentation of interaction types like DNIs and DAIs, which are often missing in datasets despite their clinical relevance.

**Fig. 8:**
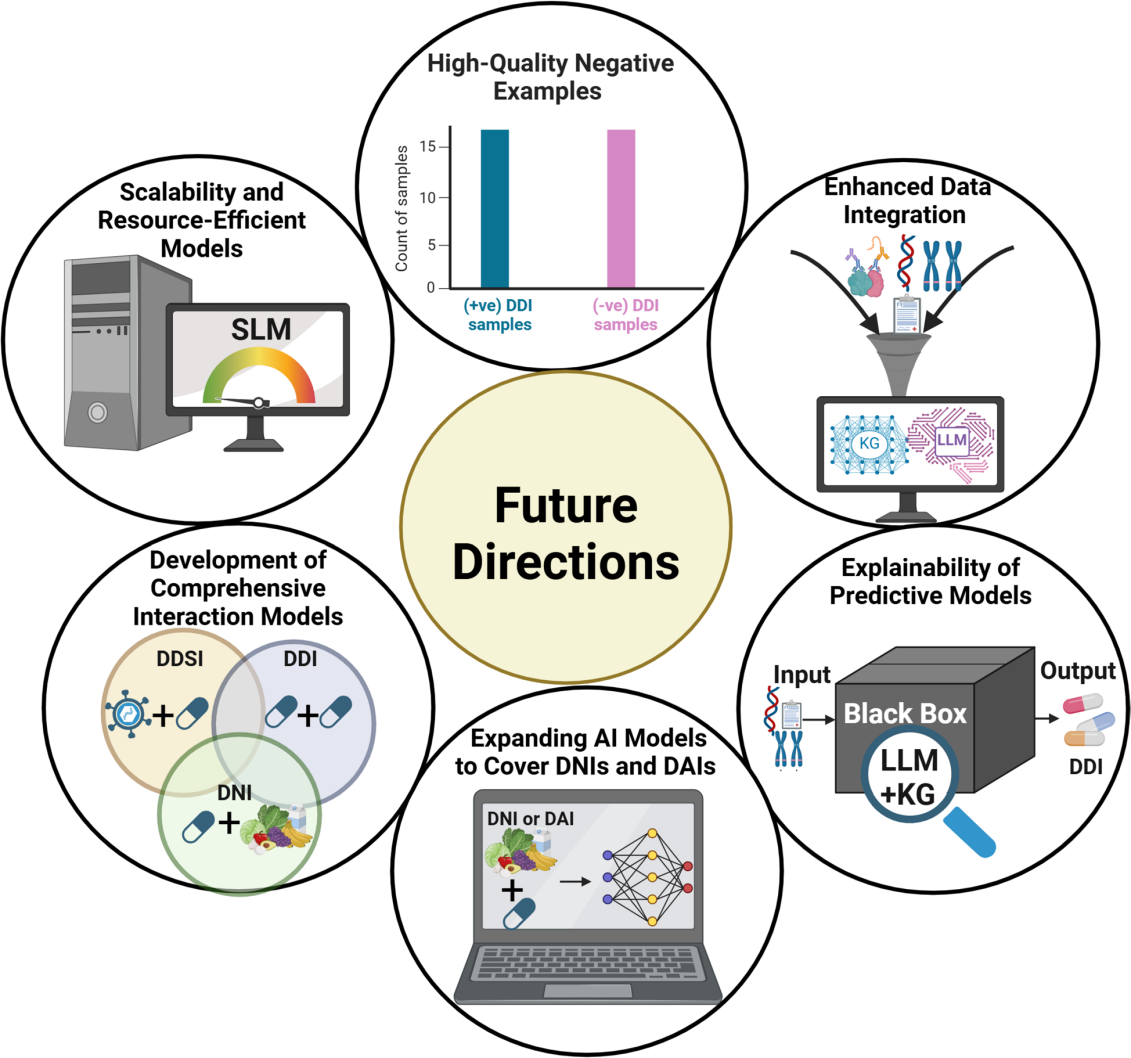
Future directions in AI-driven drug interaction prediction. Created with BioRender.com

These challenges can be addressed by combining Large Language Models (LLMs) and KGs [190]. LLMs process unstructured and noisy data effectively, while KGs provide structured validation, bridging data gaps and reducing biases. This synergy ensures robust data integration and enhances model explainability by grounding predictions in trustworthy knowledge [191]. To improve explainability, explainable AI approaches such as SHapley Additive exPlanations, Local Interpretable Model-Agnostic Explanations, counterfactual explanations, and attention mechanisms have emerged to address this gap by attributing predictions to biologically or clinically meaningful features [192]. Importantly, KGs enhance explainability by embedding biomedical knowledge into the model and enabling path-based reasoning that can be traced and interpreted [193]. Meanwhile, LLMs offer a promising direction for enhancing explainability by translating complex model outputs into human-understandable narratives, addressing the black-box nature of deep learning systems and increasing user trust [194]. Advancing from opaque models to transparent, clinically grounded systems is essential for real-world adoption. By complementing LLMs, KGs validate outputs, improve reasoning, and support transparent decision-making—critical for high-stakes applications like healthcare (Fig. 8).

Resource and scalability constraints limit the use of advanced models in constrained environments. While LLMs and KGs are effective, Small Language Models (SLMs) offer a practical, efficient alternative [195]. SLMs run on devices with low RAM requirements, enabling local real-time processing, and ensuring strong data privacy. Their fast, low-latency inference and lack of cloud dependency make them ideal for mobile, edge, and real-time applications. SLMs are easily fine-tuned for domains like healthcare or law and often outperform general LLMs in such settings. They are well-suited for tasks like API calls, mobile control, and medical emergencies, while also reducing costs and addressing privacy concerns [196, 197]. Future research should explore integrating genomics (e.g., genome, transcriptome, epigenome), clinical, and microbiome data alongside existing AI-driven approaches. This multimodal integration—supported by KGs and LLMs—could improve predictive accuracy, enable more nuanced contextual understanding, and advance personalized medicine in drug interaction modeling [10].

To address the underrepresentation of DNIs and DAIs, future research must enrich datasets and develop specialized algorithms. DNIs—such as DFIs, DMIs, and DSIs—are often overlooked despite their clinical impact [161, 167, 186, 189]. Similarly, predicting DAIs is hindered by privacy-restricted clinical data, incomplete datasets, and the absence of specialized algorithms [155]. Both require diverse AI models using genetic, biochemical, and molecular data to enhance accuracy. Comprehensive models that map high-order interactions between drugs, nutrients, and diseases are crucial for improving prediction and advancing personalized medicine.

## Conclusion

This systematic review presents the first comprehensive taxonomy of AI-driven models for predicting diverse drug interactions—including DDIs, DDSIs, and DNIs—by synthesizing methodologies, model types, and challenges across 147 studies published between 2018 and 2024. In addition to outlining key machine learning, deep learning, and graph-based models, we emphasize the transformative role of Large Language Models (LLMs) and knowledge graphs (KGs), particularly when integrated with explainable AI techniques such as SHAP, LIME, and attention mechanisms to enhance clinical interpretability. Despite these advances, substantial gaps remain in underrepresented interaction types like DNIs and DAIs, where both predictive performance and transparency are still limited. Future research should focus on combining KGs with LLMs or resource-efficient Small Language Models (SLMs), supported by robust explainable AI frameworks, to enable scalable and clinically trustworthy predictions. This integration is especially crucial for modeling high-order, overlapping interactions that span multiple biological systems. Furthermore, incorporating domain-specific knowledge and graph-based reasoning can unlock deeper insights into the complex, interconnected nature of drug interactions. By addressing data heterogeneity, limited explainability, and overlooked interaction types, this review lays the groundwork for next-generation AI tools that not only anticipate adverse interactions but also deliver actionable, personalized insights to improve drug safety and therapeutic precision in real-world clinical settings.

## Supplementary Information

Supplementary Material 1.

Supplementary Material 2.

## Abbreviations

AI: Artificial intelligence
AUC: Area under the curve
BiLSTM: Bidirectional long short-term memory
BioBERT: Bidirectional Encoder Representations from Transformers for Biomedical Text Mining
CNNs: Convolutional neural networks
DAIs: Drug–allergy interactions
DDIs: Drug–drug interactions
DDSIs: Drug–disease interactions
DFIs: Drug–food interactions
DHIs: Drug–herb interactions
DL: Deep learning
DMIs: Drug–microbiome interactions
DNIs: Drug–nutrient interactions
DNNs: Deep Neural Networks
DSIs: Drug–supplement interactions
EHRs: Electronic Health Records
FDA: Food and Drug Administration
GBM: Graph-based method
GCNs: Graph Convolutional Networks
GIP: Gaussian interaction profile
GNNs: Graph Neural Networks
KGs: Knowledge graphs
LLMs: Large Language Models
MF: Matrix factorization
ML: Machine learning
NLP: Natural language processing
PRISMA: Preferred Reporting Items for Systematic Reviews and Meta-Analyses
SVM: Support vector machine

## Declarations

### Data and materials

All data used during this study are included in this published article (and its supplementary information files).
